# Pulmonary artery catheter use and in-hospital outcomes in cardiac surgery: a systematic review and meta-analysis

**DOI:** 10.1093/icvts/ivae129

**Published:** 2024-07-08

**Authors:** Lisa Q Rong, Grant Luhmann, Antonino Di Franco, Arnaldo Dimagli, Luke A Perry, Andrew P Martinez, Michelle Demetres, C David Mazer, Rinaldo Bellomo, Mario Gaudino

**Affiliations:** Department of Anesthesiology, Weill Cornell Medicine, New York, NY, USA; Department of Anesthesiology, Weill Cornell Medicine, New York, NY, USA; Department of Cardiothoracic Surgery, Weill Cornell Medicine, New York, NY, USA; Department of Cardiothoracic Surgery, Weill Cornell Medicine, New York, NY, USA; Department of Anaesthesia and Pain Management, Royal Melbourne Hospital, Melbourne, VIC, Australia; Department of Critical Care, University of Melbourne, Melbourne, VIC, Australia; Department of Anesthesiology, Weill Cornell Medicine, New York, NY, USA; Samuel J. Wood Library and C.V. Starr Biomedical Information Centre, Weill Cornell Medicine, New York, NY, USA; Departments of Anaesthesia and Critical Care, Keenan Research Centre for Biomedical Science and Li Ka Shing Knowledge Institute of St Michael's Hospital, Toronto, Ontario, Canada; Department of Anesthesia, University of Toronto, Toronto, ON, Canada; Department of Critical Care, University of Melbourne, Melbourne, VIC, Australia; Department of Intensive Care, Royal Melbourne Hospital, Melbourne, VIC, Australia; Australian and New Zealand Intensive Care Research Centre, Monash University, Melbourne, VIC, Australia; Department of Cardiothoracic Surgery, Weill Cornell Medicine, New York, NY, USA

**Keywords:** Cardiac surgery, Meta-analysis, Mortality, Perioperative outcomes, Pulmonary artery catheter

## Abstract

**OBJECTIVES:**

To determine the association of intraoperative pulmonary artery catheter (PAC) use with in-hospital outcomes in cardiac surgical patients.

**METHODS:**

MEDLINE, Embase, and Cochrane Library (Wiley) databases were screened for studies that compared cardiac surgical patients receiving intraoperative PAC with controls and reporting in-hospital mortality. Secondary outcomes included intensive care unit length of stay, cost of hospitalization, fluid volume administered, intubation time, inotropes use, acute kidney injury (AKI), stroke, myocardial infarction (MI), and infections.

**RESULTS:**

Seven studies (25 853 patients, 88.6% undergoing coronary artery bypass graft surgery) were included. In-hospital mortality was significantly increased with PAC use [odds ratio (OR) 1.57; 95% confidence interval (CI) 1.12–2.20, *P* = 0.04]; PAC use was also associated with greater intraoperative inotrope use (OR 2.61; 95% CI 1.54–4.41) and costs [standardized mean difference (SMD) = 0.20; 95% CI 0.16–0.23], longer intensive care unit stay (SMD = 0.29; 95% CI 0.25–0.33), and longer intubation time (SMD = 0.44; 95% CI 0.12–0.76).

**CONCLUSIONS:**

PAC use is associated with significantly increased odds of in-hospital mortality, but the amount and quality of the available evidence is limited. Prospective randomized trials testing the effect of PAC on the outcomes of cardiac surgical patients are urgently needed.

## Graphical Abstract

**Figure:**
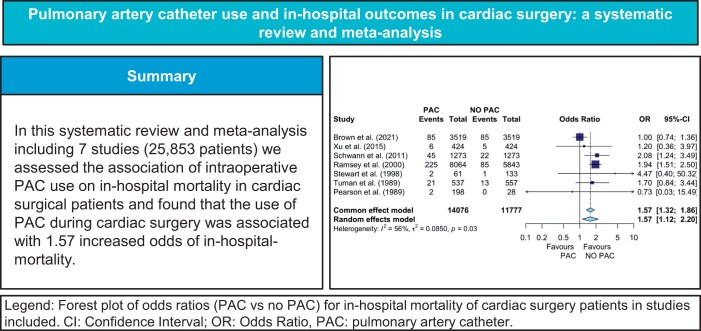

## INTRODUCTION

The use of pulmonary artery catheters (PACs) in intensive care, non-cardiac major surgery and acute coronary syndrome patients has been well-studied, and enthusiasm for its use has waned due to equivocal benefit, or evidence suggesting PAC use was associated with worse outcomes. However, PACs are still used in over one-third of all cardiac surgeries [1–3]. Cardiac surgical patients, many with significant cardiac disease, may benefit from PAC use and goal-directed therapy, given the dynamic environment of the cardiac operating room, acute cardiac insults from cardiopulmonary bypass and aortic cross clamping, and large fluid shifts leading to increased risk of haemodynamic instability and cardiac dysfunction. Additionally, intraoperative PAC use by cardiac anaesthesiologists familiar with interpreting its values in a goal-directed dynamic environment may lead to higher potential benefit of PAC use. However, the question of the potential benefit of PAC use in cardiac surgery remains largely unsolved [4–6].

In this systematic review and meta-analysis, we evaluated the association of intraoperative PAC use with in-hospital mortality and other outcomes in cardiac surgical patients. We hypothesized that PAC use would be associated with decreased in-hospital mortality because of improved haemodynamic optimization with more information such as cardiac output, pulmonary artery pressures and systemic vascular resistance. Secondary outcomes included acute kidney injury (AKI), stroke, myocardial infarction (MI), length of intensive care unit (ICU) stay, hospitalization cost, fluid volume administered, intubation time, inotrope use and infections.

## MATERIALS AND METHODS

This systematic review and meta-analysis was prespecified and performed according to the Preferred Reporting Items for Systematic Reviews and Meta-Analyses (PRISMA) guidelines [7]. A medical librarian was involved in the development of the string and search strategy (M.D.). Institutional review board and ethical approval were not requested because no human or animal subjects were involved.

### Search strategy

We searched Ovid MEDLINE, Ovid Embase and the Cochrane Library (Wiley) from 1970 to August 2022 for all subject headings and associated keywords such as catheterization, Swan-Ganz, in combination with terms for open cardiac surgeries including aortic valve repair, cardiac valve annuloplasty and myocardial revascularization (full search strategy is reported in Supplementary Material, Appendix). We screened citations of potentially eligible articles without language or publication date restrictions. Two reviewers independently screened titles and abstracts to identify potentially eligible articles for full-text review. Disagreements between reviewers were resolved by consensus or a 3rd-party (L.Q.R.).

### Study selection criteria

Studies were screened and included if (1) the patient population underwent cardiac surgery with sternotomy; (2) the study design was a randomized controlled trial or comparative observational study; (3) the intervention group received intraoperative PAC and the comparator group did not and (4) in-hospital mortality was listed as one of the outcomes. Articles that were not in English were excluded. In studies utilizing multicentre databases with overlapping populations, the most recent studies were chosen.

After results were de-duplicated, a total of 2431 abstracts were screened by 2 reviewers (A.D. and G.L.). After abstract review, full texts of manuscripts were independently assessed for eligibility by the same 2 reviewers. The PRISMA flow diagram is shown in Supplementary Material, Fig. S1.

### Data extraction

Two reviewers (A.D. and G.L.) independently extracted pertinent data from included studies using data collection forms. Disagreements were resolved by consensus.

### Risk of bias assessment

Two reviewers (A.D. and G.L.) independently assessed risk of bias of the 6 included non-randomized studies using the Risk of Bias in Non-Randomized Studies—of Interventions (ROBINS-I) tool (accessed April 2023) [8]. These non-randomized studies were categorized to low, moderate or serious risk of bias within the following 7 domains: domain 1, bias due to confounding; domain 2, bias due to patient selection; domain 3, bias in classification of interventions; domain 4, bias due to deviations from intended interventions; domain 5, bias due to missing data; domain 6, bias in measurement of outcomes and domain 7, bias in selection of the reported result. The overall risk of bias for each included study was categorized as ‘low’ if the risk of bias was low in all domains, ‘moderate’ if the risk of bias was moderate or low in all domains, ‘serious’ if the risk of bias was serious but not critical in at least 1 domain or ‘critical’ if the risk of bias was critical in at least 1 domain. Risk of bias in the sole randomized study was assessed using the revised Cochrane risk-of-bias tool (RoB 2) for randomized trials (accessed January 2024) [9]. The randomized study was categorized to low, some concerns or high risk of bias on the basis of the following 5 domains: domain 1, bias arising from the randomization process; domain 2, bias due to deviations from intended intervention; domain 3, bias due to missing data; domain 4, bias in measurement of the outcome and domain 5, bias in selection of the reported result. Disagreements were resolved by consensus or 3rd party (L.Q.R.).

### Outcomes

The primary outcome was in-hospital mortality. Secondary outcomes included AKI, stroke, MI, length of ICU stay, hospitalization cost, fluid volume administered, intubation time, inotrope use and infections. Only in-hospital outcomes were used for the analysis of secondary outcomes. Long-term outcomes, if reported, were excluded in the analysis.

### Statistical analysis

Binary outcomes were extracted as the number of events in each group and pooled using an inverse variance method and reported as odds ratio (OR) and 95% confidence interval (CI). Continuity correction was applied for cells with 0 events. Continuous outcomes were extracted as mean and standard deviation and pooled using an inverse variance method and reported as standardized mean difference (SMD) and 95% CI.

Statistical heterogeneity was calculated with I^2^, with low, moderate and high heterogeneity defined as I^2^ <25%, 25% to 50% and >50%, respectively [10]. A random effects model was applied. The presence of publication bias was assessed qualitatively by visual inspection of the funnel plot and quantitatively by Egger’s test. Given the possible limitation of Funnel plot in assessing publication bias when less than 10 studies are included, Tang’s regression was also used [11].

A leave-one-out approach, where recalculation of meta-analytic estimates is done after each study exclusion, was used as a sensitivity analysis for the primary outcome. In addition, a sensitivity analysis including only propensity-matched studies only was performed. Meta-regression was performed including pertinent clinical variables such as age, sex, type of cardiac surgery, preoperative risk factors (diabetes, congestive heart failure and chronic kidney disease), study year and risk of bias.

In all analyses, the no PAC group was the reference group. Statistical analyses were performed in R version 4.0.3 (R Foundation for Statistical Computing) using the packages: ‘meta’, ‘dmetar’ and ‘robvis’.

## RESULTS

Of the 3587 studies identified, 9 met the inclusion criteria. Of these, 2 studies were excluded due to patient overlap [12, 13]. The 7 remaining studies totalled 25 853 patients, 14 076 with PACs and 11 777 without [14–20].

Overall, 22 927 (88.6%) patients underwent coronary artery bypass grafting (CABG), 1784 (6.9%) underwent valvular surgery, and 1142 (4.4%) underwent combined valve and CABG surgery. The sample size of the included studies ranged from 114 to 13 907 patients; 5 were retrospective cohort studies [14–18], with only 2 prospective trials (a prospective cohort study [19] and a randomized controlled trial) [20], and only 2 were multicentre [16, 17]. The year of publication ranged from 1989 to 2021; the study characteristics are presented in Table 1, including the risk of bias, and statistical adjustments. Variables considered for statistical adjustment in each study are reported in detail in Supplementary Material, Table S1.

**Table 1:** Characteristics of included studies

Author	Year	*N* ^a^	Design	Month/year of surgery	Bias risk^b^	Surgical population	Exclusion criteria	Comparison	Statistical adjustment^c^	Patient characteristics included in regression^d^
Brown *et al*.	2021	7038	Retrospective observational, single-centre	2010–2018	Low	CABG, single valve, CABG + single valve	Procedures not listed to the left	CVC	Propensity-matched cohorts	Prescence or absence of patient risk factors, type and year of surgery
Pearson *et al*.	1989	226	Prospective randomized, single-centre	N/A; 9-month enrolment	High	CABG, valve, CABG + valve	Non-elective surgical status	CVC	N/A	N/A
Ramsey *et al*.	2000	13907	Retrospective observational, database	1997	Serious	CABG	Emergent surgical status	No PAC	Multivariate regression	Patient age and demographics, hospital setting, urgency
Schwann *et al*.	2011	2546	Retrospective observational, database	11/1996–6/2000	Moderate	CABG	Subjects receiving TEE	No PAC	Propensity-matched cohorts	Prescence or absence of patient risk factors, type of surgery
Stewart *et al*.	1998	194	Retrospective observational, single-centre	4/1996–10/1996	Serious	Low-risk CABG	N/A; eligibility for CVC based on specific criteria	CVC	Multivariate regression	Prescence or absence of patient risk factors
Tuman *et al*.	1989	1094	Prospective observational, single-centre	N/A	Serious	CABG	Non-elective surgical status	CVC	Stratified patients into 3 levels of preoperative risk, then analysed groups separately	Presence or absence of 3 risk factors
Xu *et al*.	2015	848	Retrospective observational, single-centre	6/2012–12/2012	Moderate	CABG	Repeat surgery	No PAC	Propensity-matched cohorts	Prescence or absence of patient risk factors

a For studies using propensity-matched cohorts, *N* only includes the matched subjects.

b Per ROBINS-I tool for non-randomized, with the following possible outcomes: low, moderate, serious, or critical risk of bias. The RoB 2 tool for randomized studies had the following possible outcomes: low, some concerns, or high risk of bias.

c In retrospective studies, denotes what method, if any, the authors used to adjust for anticipated higher level of baseline illness in PAC subjects.

d See Supplementary Material, Table S1 for full patient characteristics included in studies’ propensity matching.

CABG: coronary artery bypass graft; CVC: central venous catheter; TEE: transoesophageal echocardiography.

**Figure 1:**
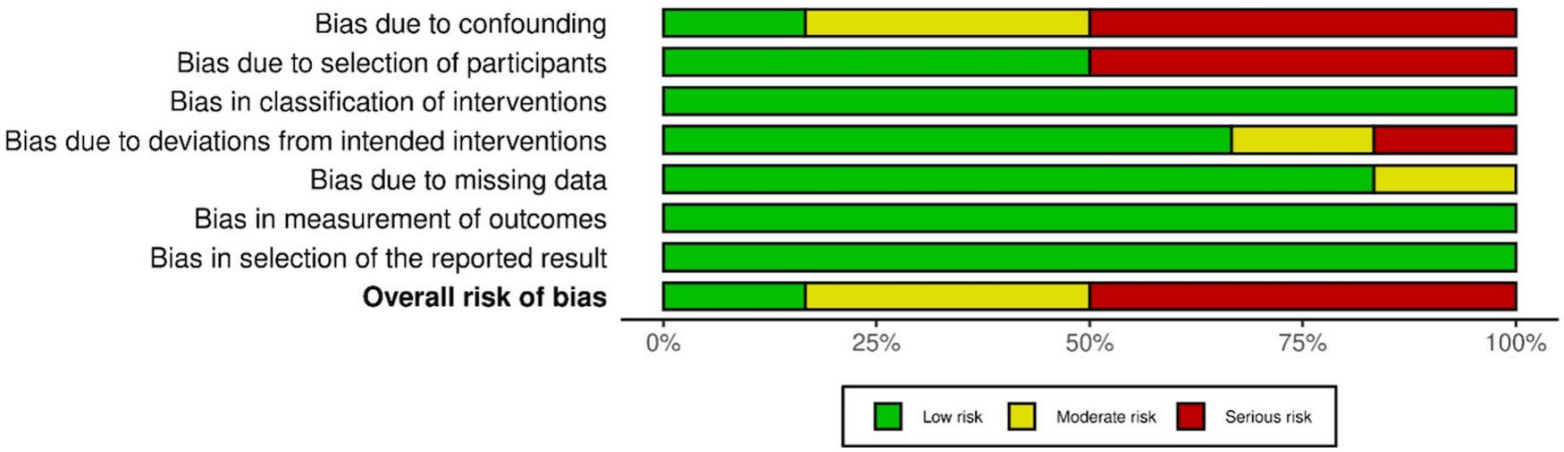
Risk of bias graph: result of authors’ assessment for each risk of bias domain showing percentages across all included studies using the ROBINS-I tool. ROBINS-I: Risk of Bias in Non-Randomized Studies—of Interventions.

**Figure 2:**
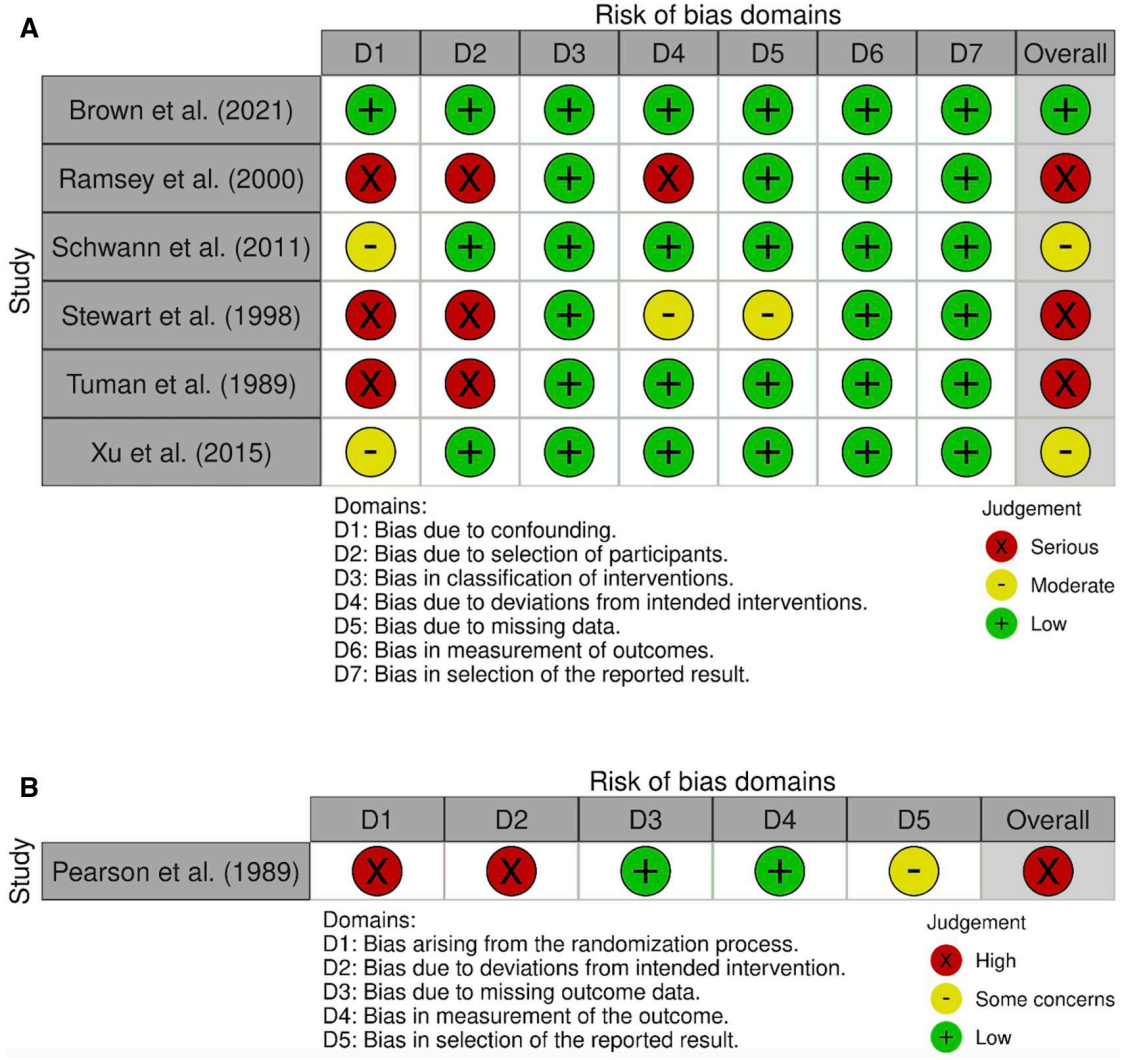
(**A**) Risk of bias summary: traffic light plot of authors’ assessment for each risk of bias domain for each study using the ROBINS-I tool. (**B**) Risk of bias summary: traffic light plot of authors’ assessment for each risk of bias domain for the sole randomized study using the RoB 2 tool. RoB 2: risk-of-bias tool; ROBINS-I: Risk of Bias in Non-Randomized Studies—of Interventions.

The risk of bias assessment is presented in Figs 1 and 2. Of the 6 non-randomized studies, 3 had a serious risk of bias according to the ROBINS-I tool, 2 had moderate risk of bias and 1 had low risk of bias. The 1 randomized study was deemed to have a high risk of bias according to the RoB 2 tool. This was due to the fact that, although patients were randomized to PAC or central venous pressure cohorts, unblinded anaesthesiologists were able to reassign patients they deemed high risk to the PAC arm preoperatively, resulting in 46 of the original 74 central venous pressure patients crossing over to the PAC arm before surgery.

Baseline patient characteristics are shown in Table 2. The mean age in each study ranged from 62 to 67 years, and the proportion of female patients ranged from 7% to 31%. The percentage of patients with diabetes, chronic kidney disease and congestive heart failure varied from 28% to 41%, 1% to 22% and 0.2% to 23%, respectively. Of the 7 studies [15–19], 5 included only CABG patients and the other 2 studies [14, 20] included patients undergoing CABG and valve surgeries.

**Table 2:** Baseline characteristics of subjects

First author, publication year	Treatment group	*n*	Age y ± SD	Female, *n* (%)	DM, *n* (%)	CKD, *n* (%)	CHF, *n* (%)	Isolated CABG, *n* (%)	Valve^a^, *n* (%)	Elective surgical status (%)
Brown, 2021	PAC	3519	68 ± 12	1091 (31.0)	1441 (40.9)	88 (2.5)	837 (23.8)	2126 (60.4)	1393 (39.5)	1589 (45.2)
	No PAC	3519	67 ± 12	1069 (30.3)	1444 (41.0)	91 (2.6)	923 (26.2)	2044 (58.1)	1475 (41.9)	1553 (44.1)
Pearson, 1989	PAC	198	NA	NA	NA	NA	NA	142 (71.7)	56 (28.3)	NA
	No PAC	28	NA	NA	NA	NA	NA	26 (92.9)	2 (7.1)	NA
Ramsey, 2000	PAC	8064	NA	2299^b^	NA	NA	NA	8064 (100)	0	4230^b^
	No PAC	5843	NA	157^b^	NA	NA	NA	5843 (100)	0	377^b^
Schwann, 2011	PAC	1273	64.3 ± 10.0	273 (7.1)	362 (28.4)	NA	NA	1273 (100)	0	NA
	No PAC	1273	64.7 ± 10.1	266 (20.1)	350 (27.5)	NA	NA	1273 (100)	0	NA
Stewart, 1998	PAC	61	67.5 ± 9.3	25 (41.0)	21 (34.4)	NA	8 (13.1)	61 (100)	0	15 (24.6)
	No PAC	133	63.3 ± 10.7	54 (40.6)	40 (30.1)	NA	2 (1.5)	133 (100)	0	47 (35.3)
Tuman, 1989	PAC	537	62.7 ± 10.3	114 (21.2)	132 (24.6)	121 (22.5)	126 (23.4)	537 (100)	0	NA
	No PAC	557	62.6 ± 10.0	140 (25.1)	156 (28.0)	110 (19.7)	75 (13.5)	557 (100)	0	NA
Xu, 2015	PAC	424	62.4 ± 9.2	114 (26.9)	136 (32.1)	9 (2.1)	3 (0.7)	424 (100)	0	NA
	No PAC	424	63.1 ± 7.8	90 (21.2)	121 (28.5)	5 (1.2)	1 (0.2)	424 (100)	0	NA

a Includes isolated valvular procedures and valve/CABG combined surgery.

b Percentages not calculated as subject characteristics are only available for a portion of the no PAC group.

CHF: congestive heart failure; CKD: chronic kidney disease; DM: diabetes mellitus; SD: standard deviation.

### Primary outcome

**Figure 3:**
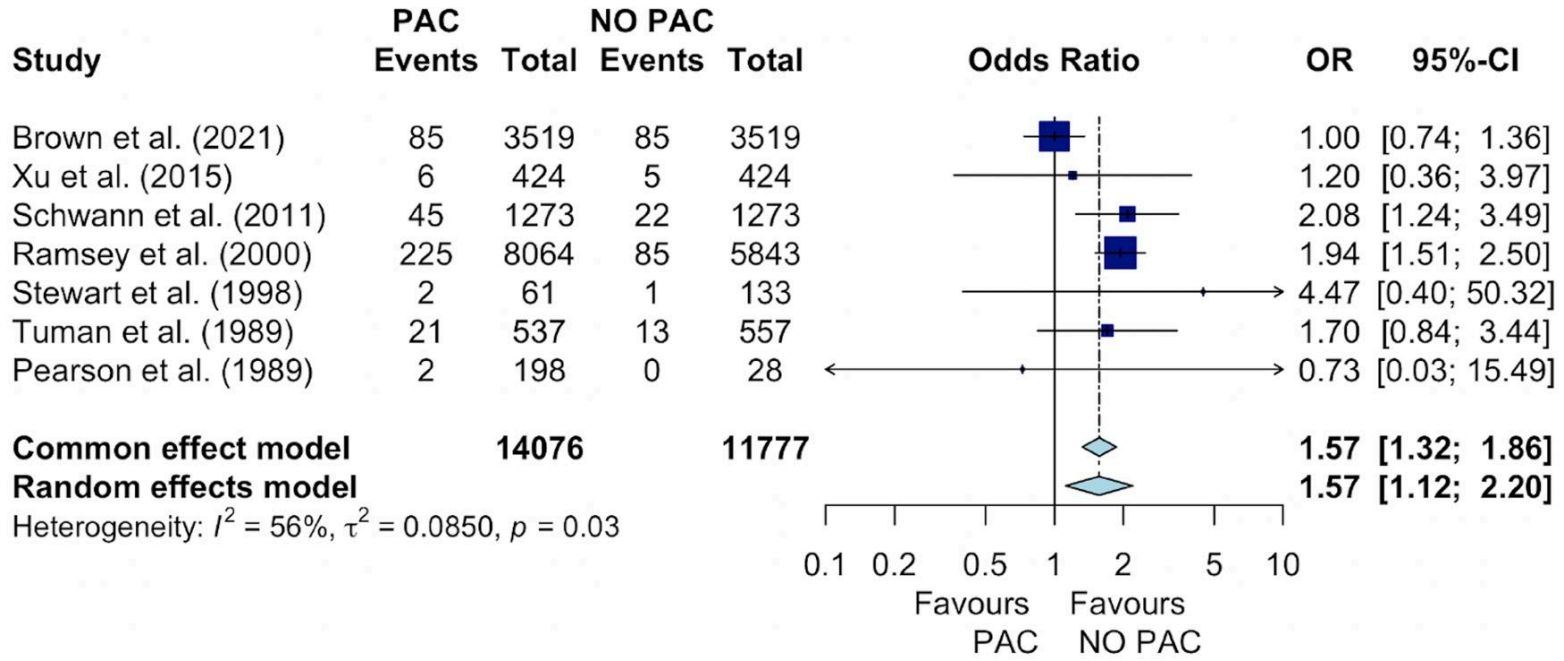
Forest plot of odds ratios (PAC vs no PAC) for in-hospital mortality of cardiac surgery patients. CI: confidence interval; OR: odds ratio; PAC: pulmonary artery catheter.

PAC use was associated with significantly increased odds of in-hospital mortality (OR 1.57; 95% CI 1.12–2.20, *P *=* *0.04) as shown in Fig. 3. This finding remained consistent in the leave-one-out sensitivity analyses (Supplementary Material, Fig. S2A) and was qualitatively consistent in propensity-matched studies (Supplementary Material, Fig. S2B).

At meta-regression, we found that the higher the proportion of CABG patients, the higher the risk of mortality was in the PAC group, while the higher the proportion of diabetic patients and of elective surgery the lower the risk of mortality in the PAC group; the mortality in the PAC group was significantly lower in the most recent studies (Table 3).

**Table 3:** Meta-regression on primary outcome of mortality

Variable	Estimates	95% CI	*P*-value
Study year	−0.0221	−0.0441 to −0.0001	0.048
Age	−0.1246	−0.2806 to 0.0314	0.12
Male sex	−0.0062	−0.0337 to 0.0214	0.66
Diabetes	−0.0472	−0.0843 to −0.0101	0.013
CKD	0.0278	−0.0131 to 0.0687	0.18
CHF	−0.0221	−0.0701 to 0.0258	0.37
CABG	0.0154	0.0065 to 0.0244	<0.001
Valve	−0.0268	−0.0419 to −0.0117	<0.001
Elective	−0.0586	−0.0928 to −0.0244	<0.001
Risk of bias	0.3503	−0.2926 to 0.9932	0.29

CABG: coronary artery bypass graft; CHF: congestive heart failure; CI: confidence interval; CKD: chronic kidney disease.

The funnel plot and Egger’s test did not reveal evidence of publication bias or small-study effect (*P* = 0.93) (Supplementary Material, Fig. S3). Tang’s regression did not show publication bias (*P* = 0.73).

### Secondary outcomes

**Figure 4:**
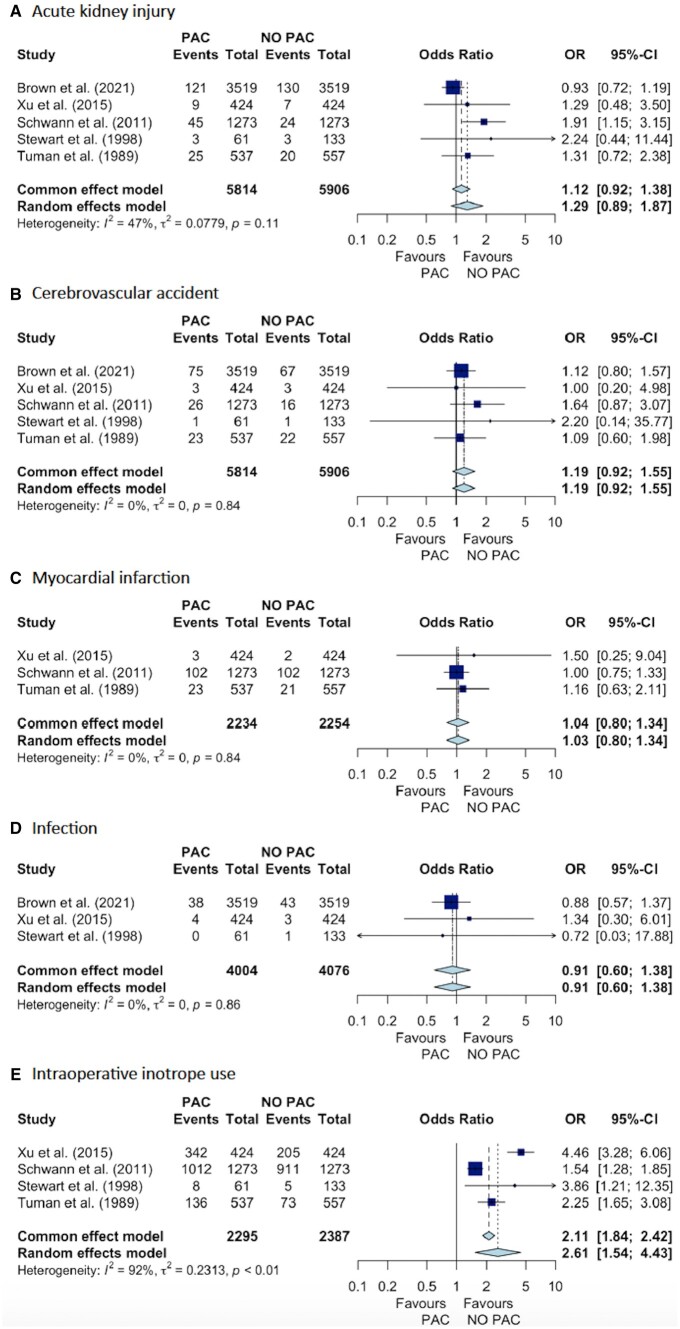
Forest plot of odds ratios (PAC vs no PAC) of secondary outcomes for (**A**) acute kidney injury, (**B**) cerebrovascular accident, (**C**) myocardial infarction, (**D**) infections, and (**E**) intraoperative inotrope use in cardiac surgery patients. CI: confidence interval; OR: odds ratio; PAC: pulmonary artery catheter.

**Figure 5:**
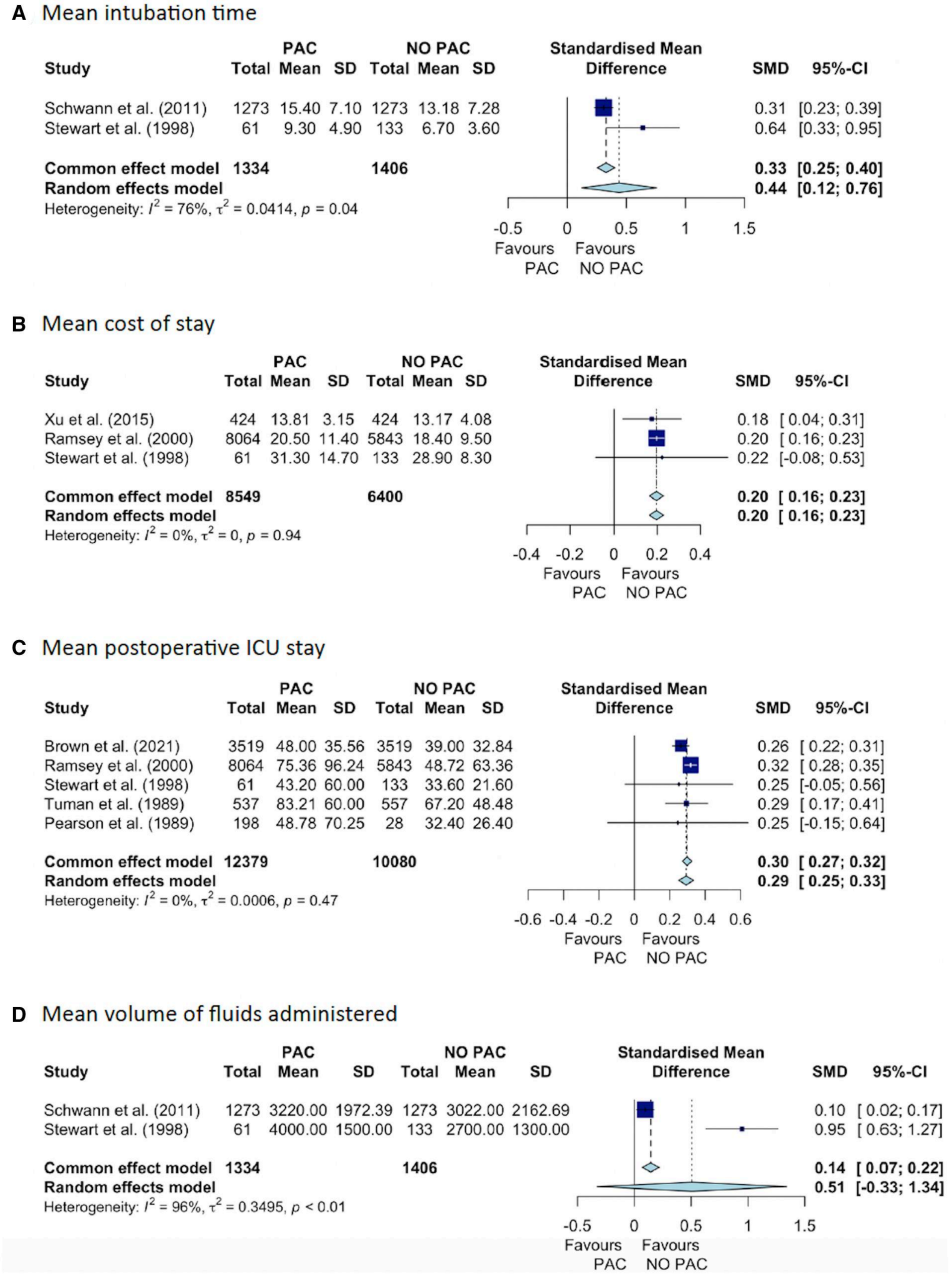
Forest plots (PAC vs no PAC) of SMD for (**A**) mean intubation time in cardiac surgery patients, (**B**) mean cost of stay, (**C**) mean postoperative ICU stay, and (**D**) mean volume of fluids administered. CI: confidence interval; ICU: intensive care unit; PAC: pulmonary artery catheter; SD: standard deviation; SMD: standardized mean difference.

PAC use was associated with significantly increased odds of intraoperative inotrope use (4 studies, OR 2.61; 95% CI 1.54–4.41), longer ICU stay (5 studies, SMD = 0.29; 95% CI 0.25–0.33), greater in-hospital cost (2 studies, SMD = 0.20; 95% CI 0.16–0.23) and longer intubation time (2 studies, SMD = 0.44; 95% CI 0.12–0.76) (Figs 4 and 5). There were no significant differences between groups in fluid volume administered or odds of in-hospital MI, AKI, stroke or infections (Figs 4 and 5).

## DISCUSSION

In this meta-analysis of 7 studies including 25 853 patients (22 927 [88.6%] CABG), we found that PAC use was associated with 1.57 increased odds of in-hospital mortality in an elective, low-risk cardiac surgical population consisting largely of CABG patients. PAC use was also associated with increased odds of intraoperative inotrope use, longer ICU stay, longer intubation time and increased in-hospital costs.

This result may be surprising but may potentially be influenced by selection bias from observational studies considering sicker patients preferentially receive PAC placement. Regardless, PAC has and continues to be a mainstay of cardiac surgery to optimize haemodynamic management.

These results build upon the existing literature on the association of PAC use with increased mortality in high-risk ICU, acute coronary syndrome and decompensated heart failure patients. While multiple studies and meta-analyses have shown a lack of mortality benefit with PAC use in the general ICU population, the literature in patients with acute cardiac disease has been conflicting [21]. In the Global Use of Strategies to Open occluded coronary arteries (GUSTO) trials, PAC use was associated with increased mortality in acute coronary syndromes, although no difference was seen in the subgroup of patients with cardiogenic shock [22]. Similarly, the Evaluation Study of Congestive Heart Failure and Pulmonary Artery Catheterization Effectiveness (ESCAPE) trial of acute decompensated heart failure found a lack of mortality benefit with PAC use [1]. Current American College of Cardiology/American Heart Association guidelines support PAC use in selected heart failure patients with cardiogenic shock or respiratory failure necessitating mechanical ventilation [23].

In cardiac surgery, the 2003 American Society of Anesthesiologists practice guidelines recommended PAC use in high-risk surgical patients when balancing the risks and benefits of the invasive procedure [24]. While PAC use declined steadily from 1990 to 2015 in cardiac surgery, its use seems to have stabilized and is currently used in approximately a third of cardiac surgeries [6].

Our results may be due to the fact that PAC-guided treatment was not beneficial to the patients. For example, incomplete information from the PAC may have led to potentially harmful interventions. A study of 1565 patients with cardiogenic shock including 8 tertiary centres compared mortality between patients with full haemodynamic PAC data, partial PAC data and no PAC data and found that patients with a complete PAC data had a significantly lower mortality overall, and in each subgroup of cardiogenic shock than the other 2 groups [25]. Correct data interpretation is also critical. In a study of 417 critical care physicians, after a real-world clinical situation with PAC data, only about one-third of physicians suggested the same treatment as experts, and one-third suggested potentially harmful therapies [26]. We found that patients who were managed with PAC received more intraoperative vasoactive drugs such as dopamine, epinephrine and milrinone. Some data support the notion that the use of inotropes is associated with increased morbidity and mortality [27–30]. Therefore, higher mortality in cardiac surgical patients monitored with a PAC may be related to overtreatment/misinterpretation of data, or incomplete data leading to harmful therapies, especially in a more elective, healthier population.

The finding that intraoperative PAC use is associated with increased mortality and increased cost, ICU length of stay and intubation time may also be due to treatment allocation, bias in observational studies and unmeasured confounders. Anaesthesiologists may have preferentially used PACs in sicker-appearing patients (‘eyeball’ test) despite similar clinical profiles. The only published randomized controlled trial (RCT) had a high crossover rate and was of low methodological quality. In addition, most of the included patients underwent elective CABG, which has the lowest mortality of cardiac surgeries and a relatively healthier patient population where PAC optimization may be less beneficial.

PAC placement itself is associated with complications, with older studies on PAC placement in cardiogenic shock quoting complication rates up to 10%, including infection, dysrhythmias and lung perforation [4]. Though rare, PAC complications include pulmonary artery perforation, rupture and pseudoaneurysm formation with mortality rates between 30% and 70% [5]. However, this is unlikely to be the major cause of our findings. Intraoperative PAC placement in cardiac surgery occurs in a controlled setting, with transoesophageal echocardiography (TEE) guidance available to potentially decrease complications and confirm correct placement [31]. Unique to the cardiac surgery operating room, intraoperative PACs are placed within central lines that are standard of care, and the complications related to insertion such as bleeding and infection may not be due to the PAC alone, but central line placement.

While major complication rates of PAC may be lower in the operating room, minor complications such as arrhythmia and coiling occur commonly, and PAC use needs to be justified beyond dogma and tradition [32]. Based on the current evidence, intraoperative PACs should be used judiciously, as it is an invasive procedure and requires increased time and resources to insert, remove and interpret. If the heart failure example is followed, benefits were seen only in the sickest patients including those that were in cardiogenic shock or intubated.

The meta-regression results suggest that study date is negatively associated with mortality. This may be due to increased anaesthesiologist experience in PAC placement in the past, when every patient received a PAC, compared to contemporary practice of PAC use in mostly sicker, high-risk cardiac surgical patients, as well as the overall decline in PAC use in cardiac surgery. Time has potentially changed PAC use in the cardiac operating room; the emergence of intraoperative TEE use after 2005 assisted in haemodynamic decision-making in the operating room beyond the PAC. PACs today have softer tips and can provide more information than in the past such as continuous cardiac output, mixed venous oxygenation, calculated stroke volume, etc. depending on the type of PAC, all of which may affect its risk/benefit ratio.

### Limitations

Our findings should be considered within the context of study limitations. Because most of the studies included were non-randomized and of variable quality, PAC treatment allocation bias and unmeasured confounding (such as effect or use of TEE) may have influenced the results. Pooled studies were mostly of CABG patients, and the results may not be generalizable to high-risk patients and cardiac surgeries. There was considerable study heterogeneity in how the PAC was used to guide haemodynamic management both within and between studies. Because the studies used different statistical adjustments, some of the event rates were adjusted for baseline characteristics, while others were unadjusted; however, the sensitivity analysis including only propensity-matched studies confirmed our primary findings. Finally, subgroup and secondary comparisons were exploratory and could have been underpowered, and some of the statistical differences may not be clinically meaningful.

## CONCLUSIONS

This systematic review and meta-analysis of 7 studies including 25 853 patients, mostly undergoing CABG, found that use of PAC was associated with significantly increased odds of in-hospital mortality after cardiac surgery. Subgroup analysis demonstrated that PAC use was also associated with increased in-hospital costs, intubation time and increased ICU length of stay, while there was no difference in the other secondary outcomes. These results question the routine use of PAC in low-risk cardiac surgery such as CABG and emphasize the need for large-scale prospective randomized trials on the association of PAC with cardiac surgical outcomes to assist with clinical decision-making.

## Supplementary Material

ivae129_Supplementary_Data

## Data Availability

The data underlying this article will be shared on reasonable request to the corresponding author.

## ABBREVIATIONS

AKI: Acute kidney injury
CABG: Coronary artery bypass grafting
CI: Confidence interval
ICU: Intensive care unit
MI: Myocardial infarction
OR: Odds ratio
PAC: Pulmonary artery catheter
ROBINS-I: Risk of Bias in Non-Randomized Studies—of Interventions
SMD: Standardized mean difference
TEE: Transoesophageal echocardiography
