# Supplementary material for: Pulmonary artery catheter use and in-hospital outcomes in cardiac surgery: a systematic review and meta-analysis
Source: Interdiscip Cardiovasc Thorac Surg. 2024 Jul 8;39(1):ivae129. doi: 10.1093/icvts/ivae129 (PMC11254303; doi:10.1093/icvts/ivae129)
Supplement: ivae129_Supplementary_Data [file ivae129_supplementary_data.docx]

**Supplementary Fig. 1.** Preferred Reporting Items for Systematic Reviews and Meta-Analyses (PRISMA) flow diagram of screened studies.


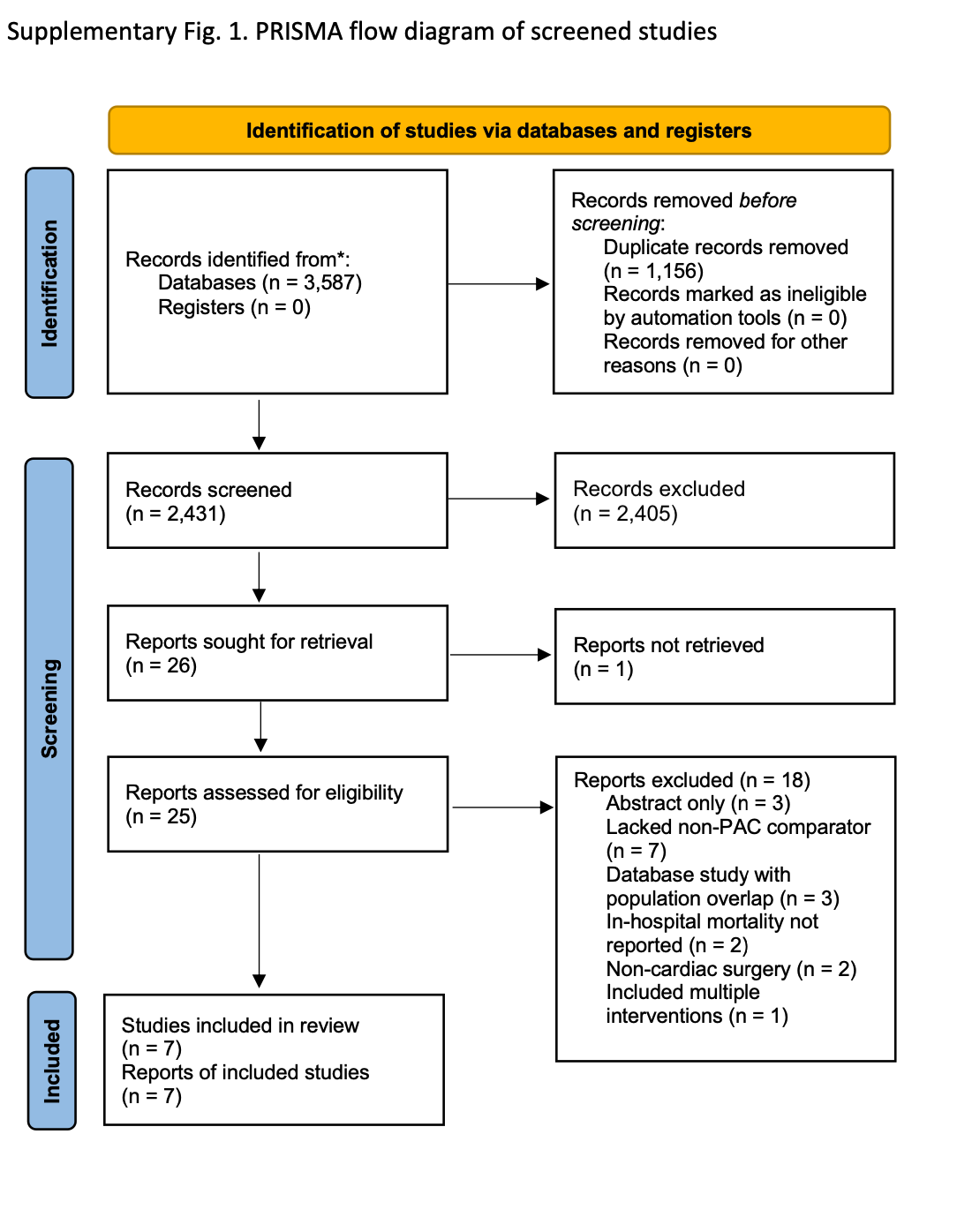


**Supplementary Fig. 2.** Sensitivity Analyses. (A) Forest plot of odds ratios (PAC vs no PAC) for in-hospital mortality of cardiac surgery patients for leave-one-out analysis and (B) forest plot of propensity-matched studies.


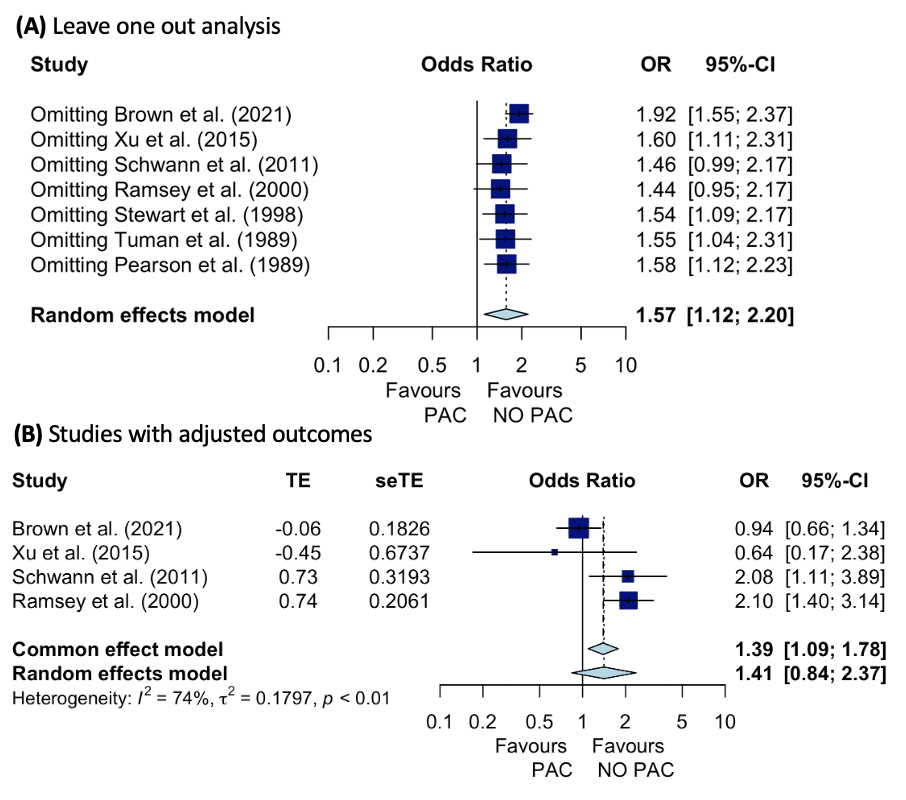


**
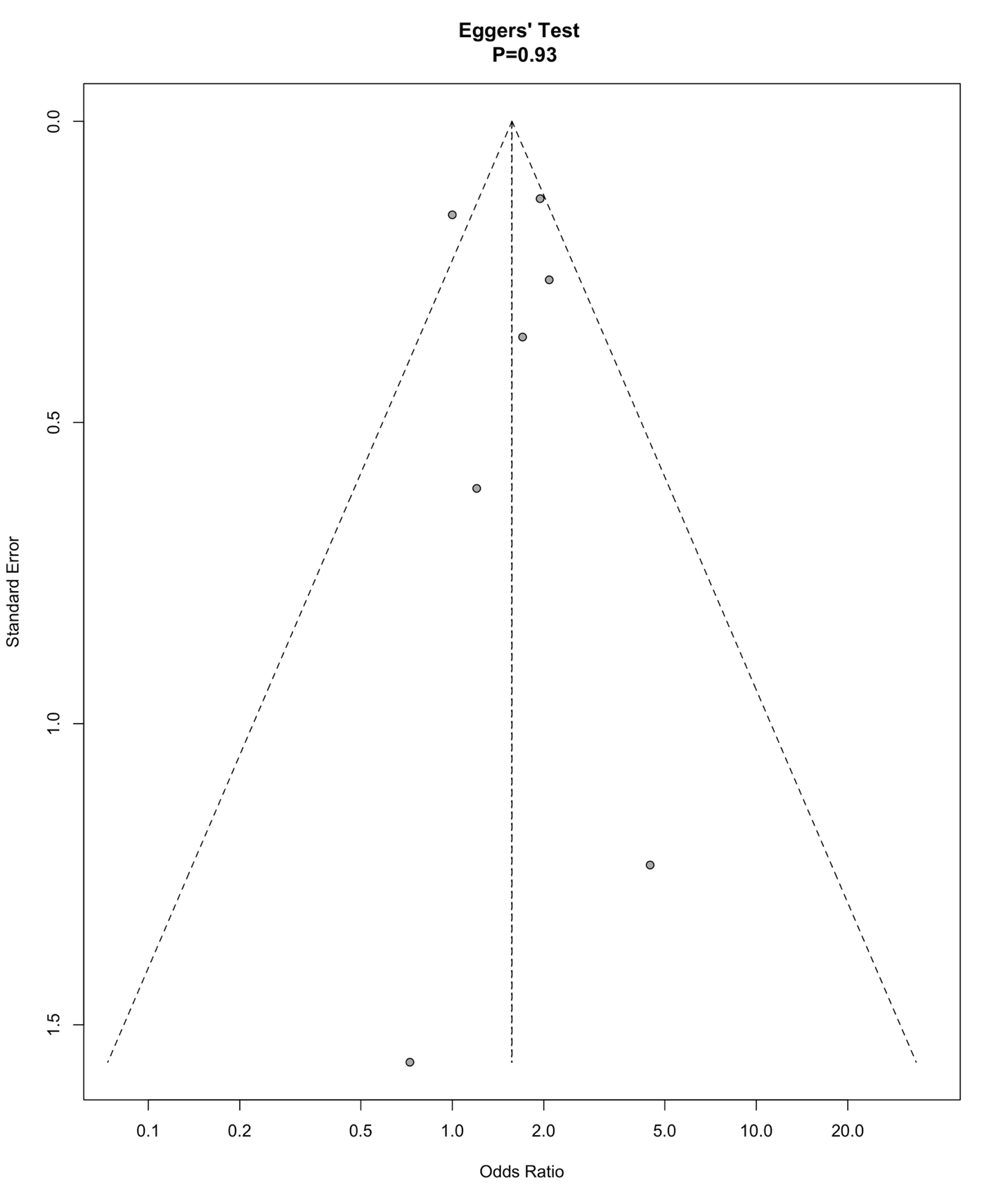
Supplementary Fig. 3.** Funnel plot detailing Egger’s test for bias analysis.

**Supplementary Table 1.** Patient characteristics included in propensity matching and regression models used to match baseline levels of operative risk between PAC and no PAC patients.

| \| Author \| Year \| N* \| Statistical Adjustment* \| Clinical covariates used to match preoperative risk between cohorts \| \| --- \| --- \| --- \| --- \| --- \| \| Brown et al. \| 2021 \| 7038 \| Propensity matched cohorts \| Median age, sex, race, body mass index, diabetes mellitus, chronic dialysis use, hypertension, severe chronic lung disease, peripheral vascular disease, prior cerebrovascular accident, preoperative hematocrit, preoperative creatinine, atrial fibrillation or flutter < 14 days, previous myocardial infarction < 21 days, congestive heart failure < 14 days, preoperative inotrope < 48 hours, preoperative intra-aortic balloon pump, ejection fraction, redo cardiac surgery, STS-PROM median, valve disease (aortic insufficiency, aortic stenosis, mitral insufficiency, mitral stenosis, tricuspid insufficiency), surgical status (elective, urgent, or emergency/salvage), surgical procedure (isolated CABG, isolated valve, CABG + single valve), year of index operation (2010-2014, 2015-2018) \| \| Ramsey et al. \| 2000 \| 13907 \| Multivariate regression \| Age, sex, race, admission status (urgent vs. elective), health insurance status and type, teaching hospital status, hospital setting (rural vs. urban), number of CABG operations performed at the hospital, severity of illness as assessed by the All Patient Related-Diagnosis Related Groups (APR-DRG) \| \| Schwann et al. \| 2011 \| 2546 \| Propensity matched cohorts \| Age, body surface area, sex, race, diabetes mellitus, hypertension, smoking status, unstable angina, myocardial infarction, dysrhythmia, valve disease, prior percutaneous transluminal coronary angioplasty/coronary atherectomy/intracoronary stent, prior CABG, prior valve surgery, prior other cardiac surgery, prior noncardiac surgery, neurological dysfunction, extracardiac arteriopathy, pulmonary disease, liver disease, gastrointestinal disease, renal disease, peripheral vascular disease, diabetes mellitus, anemia, left-ventricular dysfunction (moderate vs. severe), intra-aortic balloon pump (IABP), medication of inotropes/vasoconstrictors, Serum creatinine > 200 *µ*mol/L, critical state, myocardial infarction < 90 days, congestive heart failure at admission/preoperative, emergent surgical status, and concurrent valve, aortic or other combined cardiac surgery \| \| Stewart et al. \| 1998 \| 194 \| Multivariate regression \| Sex, hypertension, peripheral vascular disease, history of prior myocardial infarction, congestive heart failure, diabetes mellitus \| \| Tuman et al. \| 1989 \| 1094 \| Stratified patients into 3 levels of preoperative risk, then analyzed groups separately \| Presence or absence of 3 risk factors: Preoperative myocardial infarction < 6 weeks before admission, evidence of ventricular dysfunction, congestive heart failure. Group I patients had none of these risk factors, group II had one risk factor, and group III had two or more \| \| Xu et al. \| 2015 \| 848 \| Propensity matched cohorts \| Age, sex, body mass index, diabetes mellitus, hypertension, hyperlipidemia, regular smoking, drinking, history of renal failure, chronic liver disease, chronic obstructive pulmonary disease, peripheral vascular disease, cerebral events, myocardial infarction, chronic heart failure, atrial fibrillation, intra-aortic balloon pump, three-vessel disease, ventricular aneurysm, main stem disease, pulmonary arterial hypertension, ejection fraction, left ventricular end-diastolic diameter \| |
| --- | --- | --- | --- | --- | --- | --- | --- | --- | --- | --- | --- | --- | --- | --- | --- | --- | --- | --- | --- | --- | --- | --- | --- | --- | --- | --- | --- | --- | --- | --- | --- | --- | --- | --- | --- |

* For studies using propensity-matched cohorts, N only includes the matched subjects
